# Supplementary figures and images for: Genetic effects of long-term captive breeding on the endangered pygmy hog
Source: PeerJ. 2021 Oct 8;9:e12212. doi: 10.7717/peerj.12212 (PMC8504462; doi:10.7717/peerj.12212)

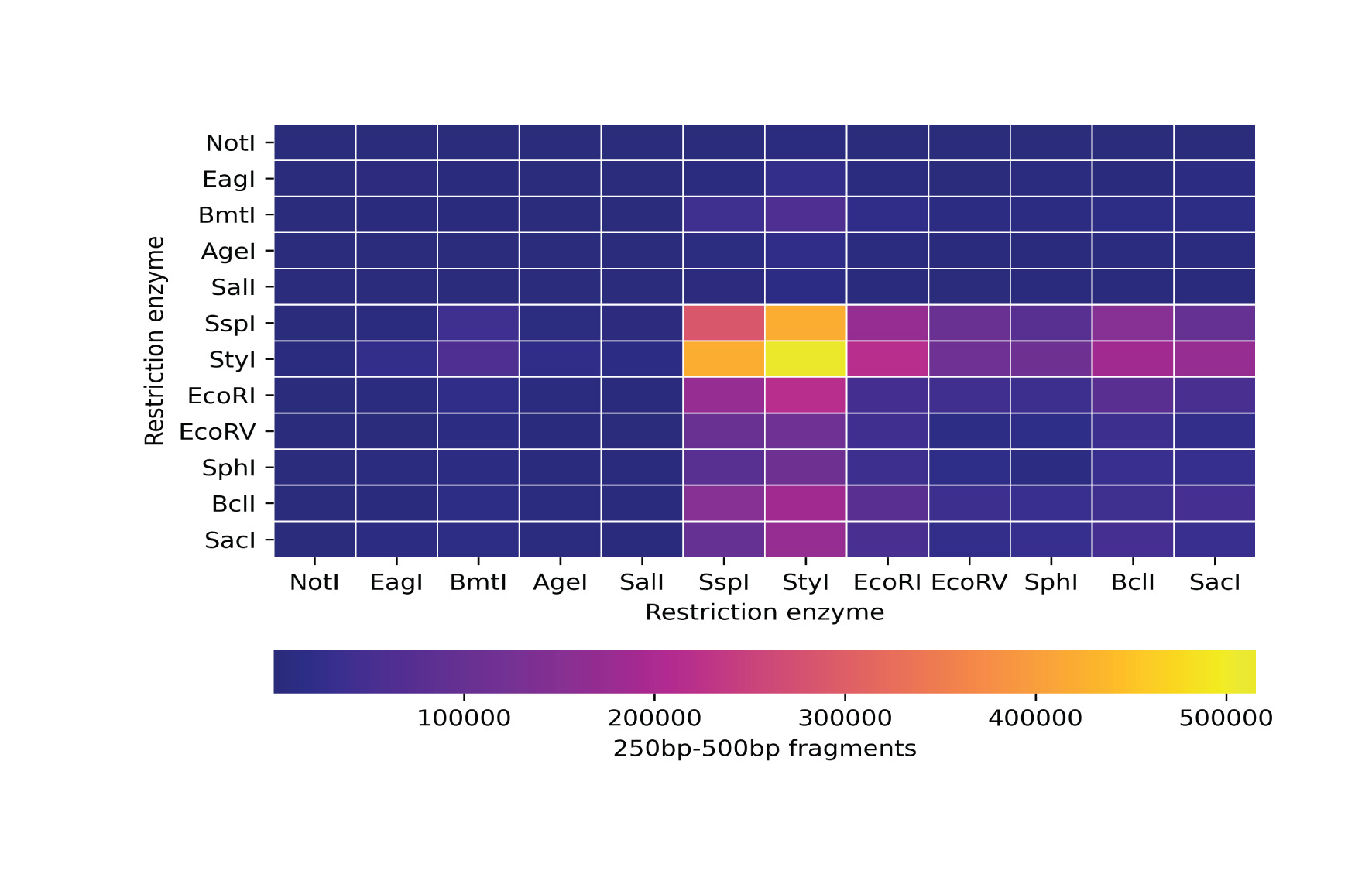

Supplement: Supplemental Information 1 [file peerj-09-12212-s001.jpg]

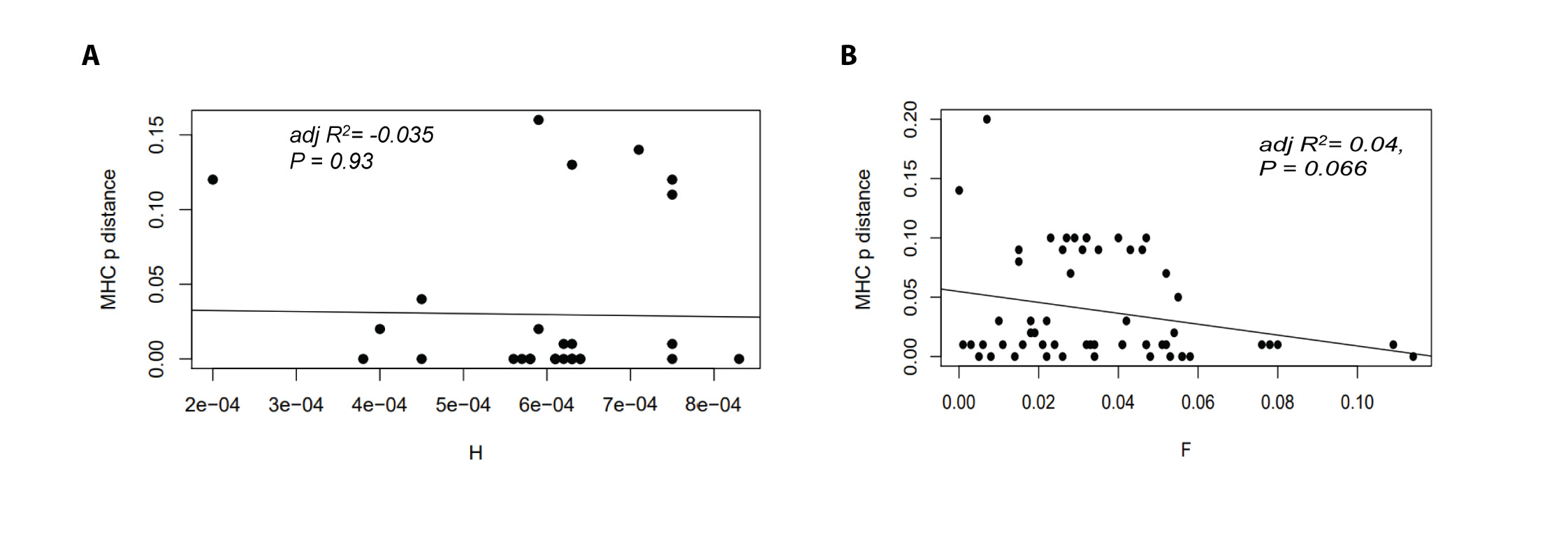

Supplement: Supplemental Information 2 [file peerj-09-12212-s002.jpg]
