# Supplementary material for: Genetic effects of long-term captive breeding on the endangered pygmy hog
Source: PeerJ. 2021 Oct 8;9:e12212. doi: 10.7717/peerj.12212 (PMC8504462; doi:10.7717/peerj.12212)
Supplement: Supplemental Information 4 [file peerj-09-12212-s004.docx]

**Genetic effects of long-term captive breeding on an endangered species: a case study on the pygmy hog**

Deepanwita Purohit^1^, Shivakumara Manu^1^, Muthuvarmadam Subramanian Ram^1,2^, Shradha Sharma^1^, Harika Patnaik^1^, Parag Jyoti Deka^3,4^, Goutam Narayan^5^ & Govindhaswamy Umapathy^1^

**Supplementary Materials and Methods**

**MHC sequence analysis and diversity estimation**

The forward and reverse MHC reads of each sample were assembled. The primer and vector sequences were removed from each consensus read. Each sequence was manually edited for any base-calling errors in CodonCode Aligner 2.0.6 (<http://www.codoncode.com/>). Reads with insertions/deletions (indels) and premature stop codons in all six reading frames were marked as pseudogenes and excluded from further analysis. The sequences were verified as MHC alleles based on their homology with the exon II region of MHC class II gene of other species in BLASTN hits. Identical sequences amplified from at least two different pygmy hogs or in two separate PCR reactions in the same pygmy hog were classified as bona fide MHC II sequence. Reads that did not pass through this step were discarded as possible PCR artefacts. All the bona fide MHC reads were aligned to form contigs and the trace sequences in the contig were cross-verified for any base calling errors at the sites of mismatches.

MHC diversity was tested using a dataset of 68 MHC sequences obtained from the study population. We computed haplotype diversity (Hd) and nucleotide diversity (π) for each group using DnaSP v.6 (Rozas et al., 2009). We used the overall mean distance between different translated MHC sequences (MHC p-distance) in each individual and the number of MHC alleles per individual as proxies for MHC diversity. Further, the parameter MHC p-distance was used to test the divergent allele advantage hypothesis in pygmy hogs (Pierini & Lenz, 2018). We used the codon-based Z test of selection in Mega 7 (Kumar et al., 2016) to test if the non-synonymous substitution rate (dN) was larger than the synonymous substitution rate (dS). We employed Tajima’s D statistics to examine the deviation from mutation-drift equilibrium and MacDonald-Kreitman test (MK test) to examine the occurrence of positive selection acting on MHC loci. For the MK test, orthologous MHC alleles from five *Sus scrofa* belonging to different populations were used as the outgroup (Table S3). Both these tests were done in DnaSp v.6.

**Mitochondrial genome assembly and analysis**

The ezRADseq strategy produces some proportion of randomly fragmented reads along with the RAD-tags. Due to the high copy number of the mitochondria, we expected to find enough randomly fragmented reads to assemble the mitochondrial genome. The raw reads were first quality trimmed and filtered using Fastp (Chen et al., 2018) with default parameters. We tried to assemble the complete circular mitochondrial genome for each of the 36 samples using GetOrganelle v1.7.2a (Jin et al., 2020). First, the mitochondrial reference genome of Pygmy hog (NC_043879.1) was used as a seed to first filter the mitochondrial reads from the nuclear reads. The filtered mitochondrial reads were then assembled *de novo* with multiple kmer lengths of 45, 65, 85, 105, 125 and then checked for the presence of mitochondrial genes. Samples with multiple fragmented contigs of mitochondria were scaffolded using RagTag (<https://github.com/malonge/RagTag>), the improved version of Ragoo (Alonge et al., 2019). The mitochondrial genomes were then brought to the same orientation as the reference genome by reverse complementation if the number of shared 35mers were greater in the reverse strand than the forward strand. We annotated the coding and non-coding regions of the mitochondria using Mitos2 (Donath et al., 2019). Finally, we re-circularised the mitochondrial genomes using MARS (Ayad et al., 2017) and aligned using Clustal Omega (Sievers et al., 2014).

**Supplementary Materials Reference**

Alonge M, Soyk S, Ramakrishnan S, Wang X, Goodwin S, Sedlazeck FJ, Lippman ZB, Schatz MC. 2019. Fast and accurate reference-guided scaffolding of draft genomes. *BioRxiv* 1:519637 DOI: 10.1101/519637.

Ayad LA, Pissis SP. 2017. MARS: improving multiple circular sequence alignment using refined sequences. *BMC genomics* 18:1-0 DOI: 10.1186/s12864-016-3477-5.

Chen S, Zhou Y, Chen Y, Gu J. 2018. fastp: an ultra-fast all-in-one FASTQ preprocessor. *Bioinformatics* 34:i884-90 [DOI: 10.1093/bioinformatics/bty560](https://doi.org/10.1093/bioinformatics/bty560).

Jin JJ, Yu WB, Yang JB, Song Y, Depamphilis CW, Yi TS, Li DZ. 2020. GetOrganelle: a fast and versatile toolkit for accurate de novo assembly of organelle genomes. *Genome biology* 21:1-31 DOI: 10.1186/s13059-020-02154-5.

Kumar S, Stecher G, Tamura K. 2016. MEGA7: molecular evolutionary genetics analysis version 7.0 for bigger datasets. *Molecular biology and evolution* 33:1870-4 [DOI: 10.1093/molbev/msw054](https://doi.org/10.1093/molbev/msw054).

Pierini F, Lenz TL. 2018. Divergent allele advantage at human MHC genes: signatures of past and ongoing selection. *Molecular biology and evolution* 35:2145-58 [DOI: 10.1093/molbev/msy116](https://doi.org/10.1093/molbev/msy116).

Rozas J. 2009. DNA sequence polymorphism analysis using DnaSP. In Bioinformatics for DNA sequence analysis pp. 337-350. Humana Press.

Sievers F, Higgins DG. 2014. Clustal Omega, accurate alignment of very large numbers of sequences. InMultiple sequence alignment methods pp. 105-116. Humana Press, Totowa, NJ.

**Supplementary figure legends**

**Figure S1**. Heatmap of DNA fragments in the range of 250-500 bp generated in different combination of restriction enzymes pairs

**Figure S2**. (a) Association plot between MHC p distance and genome-wide heterozygosity (H), (b) Association plot between MHC p distance and genome-wide kinship coefficient
